# Public awareness regarding the manufacturer provided information about medicine usage, safety, and adverse drug reactions in Balochistan, Pakistan

**DOI:** 10.3389/fphar.2023.1190741

**Published:** 2023-07-20

**Authors:** Gullab Khan, Noman Haq, Nafees Ahmad, Aqeel Nasim, Asma Javaid, Mujhammad Saood, Riffat Yasmin, Maria Tahir, Sohail Riaz, Zeeshan Danish, Ghulam Razzaq, Abdullah Khan, Muhammad Younis, Tahmina Rabbani

**Affiliations:** ^1^ Faculty of Pharmacy and Health Sciences, University of Balochistan, Quetta, Pakistan; ^2^ Provincial Drug Testing Laboratory Balochistan, Quetta, Pakistan; ^3^ Sardar Begum Dental Hospital, Peshawar, Pakistan; ^4^ Balochistan Institute of Nephrology Urology Quetta, Quetta, Pakistan; ^5^ Sardar Bahadur Khan Women’s University, Quetta, Pakistan; ^6^ Capital University of Science & Technology, Islamabad, Pakistan; ^7^ Department of Pharmacy, University of Punjab, Lahore, Pakistan; ^8^ Civil Hospital Quetta, Quetta, Pakistan

**Keywords:** public awareness, medicine information, safety, Quetta, Pakistan

## Abstract

**Objectives:** This study aimed to analyze the general public’s awareness of medicine information, safety, and adverse drug reactions in Quetta, Pakistan.

**Methods:** A cross-sectional descriptive study was conducted using random sampling from April 2020 to April 2021 in Quetta. Samples were collected from respondents who met the inclusion criteria and had visited community pharmacies. The analysis was done using SPSS version 23. Bivariate and multivariate analyses were performed to assess factors associated with good knowledge.

**Results:** Multivariate analysis revealed that purchase on prescription was a determining factor of knowledge regarding knowledge of pharmaceutical products and their provided information, medicines usage and safety, and Medication ADRs. Patients who bought medicines on prescriptions were more likely to have better knowledge. Patients having education were more likely to have better knowledge.

**Conclusion:** Public awareness about medicine information, safety, and the information provided by manufacturers is crucial to ensuring that patients have access to accurate information about their medications and can make informed decisions about their health. Healthcare providers and regulatory bodies must work together to improve access to information and promote safe medication practices.

## 1 Introduction

### 1.1 Background

Comprehensive drug regulations are essential to protecting the public from potentially dangerous or questionable medications, as they provide oversight and accountability for all aspects of pharmaceutical use within the country. In most countries, drug regulatory authorities devote more time and effort to pre-marketing than post-marketing activities (Ratanawijitrasin and Wondemagegnehu, 2002). This type of authority in Pakistan is known as the Drug Regulatory Authority of Pakistan (DRAP). Drug regulation in Pakistan was formulated in 2012. It successfully coordinates and enforces the Drug Act of 1976 (Nishtar, 2012).

Every medicine package has a patient information leaflet, a technical document that provides written information about the medication. Manufacturers give out patient information leaflets (PILs) that all have the same type of information and follow a standard format. Their major objective is to educate patients about how to take their medication, any necessary precautions, and any possible side effects (Herber et al., 2014). The package insert (PI) provides essential drug information for patients taking over-the-counter and prescription-only medications. Studies have shown that PIs help to bridge the information gap between healthcare providers and patients and enhance patients’ knowledge about medications (Dawoodi, 2016).

Numerous studies have already focused on how PILs may be improved regarding design, readability, knowledge of medicines, safety, and adverse effects. However, little is known about how patients react and behave after reading the risk information presented in PILs (Carrigan et al., 2008; Fuchs et al., 2008).

Consumers want to know more than ever about their medications and their effects to make decisions on medicine usage. Giving patients useful, well-organized leaflets that are simple to browse can result in a better quality of life, less worry, earlier detection of unpleasant side effects, and better knowledge of the treatment regimen (Fuchs et al., 2008; Julius et al., 2009). Patients with long-lasting and complicated diseases like diabetes, high blood pressure, asthma, angina, and peptic ulcers must know about their illness and how to treat it. Long-term management of these diseases entails patient self-care. Several studies have found that patients cannot control their conditions because they do not obtain the appropriate information (Morris and Halperin, 1979).

Adverse effects can be readily avoided by taking specific precautions on the patient’s part. One of these steps is ensuring that the person using the medication knows and understands everything about it. This can be done by reading the leaflet with the drug, which usually has all the important information about it. If necessary, the patient might seek clarification from the pharmacist or doctor. Because healthcare professionals are frequently quite busy, it is advised that patients prepare their questions in advance, write them down, and then submit them to their doctor or pharmacist (Alshammari, 2016).

In evaluating patients’ knowledge of medicines, the following are considered essential parameters for safe and effective use: the names of the medicines, the purpose of therapy, the duration of therapy, the dose and frequency of administration, and important side effects. Inadequate knowledge of medicines by patients may result in incorrect use, leading to treatment failure and putting the patient’s health at risk. Moreover, a lack of knowledge may cause unintended overdose or nonadherence with medicine regimens, resulting in poor outcomes, and there is no specialized marking (labeling) on medicines for illiterate patients (Mercy et al., 2006).

Patients’ knowledge of prescribed medicines is one of the most important antecedents of successful therapy. Poor knowledge about medicines can lead to serious consequences, such as nonadherence and misunderstanding of the significance of adverse events. In such resource-constrained situations, patients’ knowledge of their medications is frequently inadequate, and language problems in communication contribute to poor knowledge and medication errors. Most countries, particularly the Commonwealth, train healthcare personnel in English, and prescription labeling and discharge summaries are written in English, which most patients may not comprehend. In such environments, where patients are overcrowded, there is very little time to deliver information, even simple instructions on medication use. A prior study conducted in Sri Lanka discovered that limited English proficiency, a lower level of education, and a lack of a sense of sickness severity all reduced knowledge of recommended medications.

How thoroughly patients were informed about the medications and their safety is unknown. Furthermore, literature on consumers’ perceptions of ADR knowledge in Balochistan is scarce. This study aimed to analyze the general public’s awareness of medicine information, safety, and adverse drug reactions in Quetta, Pakistan.

## 2 Methods

### 2.1 Study design, setting, and duration

A cross-sectional descriptive study was conducted using random sampling from April 2020 to April 2021 from community pharmacies in Quetta.

### 2.1 Study population and eligibility criteria

The study population comprised people who went to community pharmacies in Quetta to get their prescriptions refilled. The survey included consumers of all ages and genders who had used drugs in the previous 3 months. Consumers under 18, who had not taken drugs in the previous 3 months and were unwilling to participate, were excluded.

### 2.3 Sampling technique and data collection

Consumers were divided into four categories: general public, students, employed, and locality. Unemployed individuals who had no education or were retired and did not want to share their income were also included in the general public. Students were targeted according to their level of studies, while employed consumers were those with any job or who were self-employed. The locality was further defined as including urban and rural consumers. Data collection began in April 2020 and was concluded in April 2021.

### 2.4 Sample size

Samples were obtained from individuals who met the predetermined inclusion criteria and had visited community pharmacies. These respondents were selected as a representative sample of individuals who seek medical advice or treatment from community pharmacies.

### 2.5 Study tool/questionnaire development and validation

A questionnaire based on the existing literature was developed and validated (Sales et al., 2017; Salgueiro et al., 2019). The final version had closed-ended questions about demographics, medicine use, how to read and understand the leaflet, and how safe people thought drugs were. Aside from the demographic and disease parts, the questionnaire comprised three sections: (Ratanawijitrasin and Wondemagegnehu, 2002): basic knowledge of pharmaceutical products and the information provided, (Nishtar, 2012), knowledge of medicines for their use and safety, and (Herber et al., 2014) knowledge of medication safety and adverse events.

The questionnaire was translated into Urdu using a forward-backward procedure by native Urdu speakers who also spoke English as a second language. The questionnaire was subjected to content, face validation, and pilot testing. According to Lawshe’s method, content validity was evaluated (Lawshe, 1975). According to the criteria, CVR ≥0.75 was acceptable for each item (Lawshe, 1975).

### 2.6 Scoring of the questionnaire

The content validity index (CVI) was 0.93, considered satisfactory. Face validation was also performed by face-to-face assessment, and certain grammatical problems were corrected. The questionnaire was distributed to nine pilot participants. There were no reports of difficulties comprehending the goods. The pilot data were excluded from the analysis.

Scoring of the Questionnaire was adopted (Islam et al., 2020). The scoring criteria of the questionnaire are documented in the Supplementary Material S1.

### 2.7 Statistical analysis

A descriptive statistic was used to produce the results of the study. Social and economic characteristics, purchase of prescription medication, and disease prevalence were described using simple frequencies. Knowledge about pharmaceutical products, including medication usage and adverse reactions, was also described using simple frequencies. We categorized participants for knowledge of pharmaceutical products and their provided information with scores equal to or greater than 8 as having “good knowledge” and those with scores equal to or below 7 as having “poor knowledge.” For knowledge of medicines for their usage and safety with scores equal to or greater than 8 as having “good knowledge” and those with scores equal to or below 7 as having “poor knowledge.” For Knowledge Regarding Medication, ADRs with scores equal to or greater than 9 as having “good knowledge,” and those with scores equal to or below 8 as having “poor knowledge.” The simple frequency of domains related to knowledge of pharmaceutical products and their provided information, knowledge of medicines for their usage and safety, and knowledge of medication ADRs were used to calculate the population. All three levels of consumer knowledge were subjected to bivariate analysis. To evaluate factors associated with “good knowledge,” a binary logistic regression analysis was performed using the dichotomous variable. Those with “good knowledge” based on the cumulative score were categorized as having “good knowledge of drugs.” Gender, age, education, occupation, location, and prescription purchase were the independent covariates. The independent co-variants were chosen based on the bivariate analyses (r = 0.15) to generate the best-fit model. The z-test was used to determine the significance of mean differences. *p* values of 0.05 were deemed statistically significant in all analyses. Those variables that showed significant associations were further tested using multiple logistic regression. SPSS Software (version 23) was used in all analysis.

### 2.8 Ethical approval

The study was approved by the ethical committee of the Faculty of Pharmacy and Health Sciences, University of Baluchistan Quetta, as per the guidelines of the National Bioethical Committee of Pakistan (N.B.C, 2016). The consent form informed all the participants that their participation was voluntary.

## 3 Result

### 3.1 Demographic characteristics

Table 1 shows demographic characteristics. Out of 1,162 respondents, the majority of respondents n = 339 (29.2%), were aged between 22 and 31 years; males were n = 994 (86.5%) and females were n = 168 (14.5%), n = 972 (83.6%) were married. The majority of respondents (21.7%) held the SSC qualification. Although many respondents, n = 384 (33.0%), were self-employed, n = 271 (23.3%) were unemployed. Most respondents, n = 446 (38.6%), were unwilling to disclose their household income. Surprisingly, the locality of most respondents, n = 646 (55.6%), was urban.

**TABLE 1:** Demographic characteristics.

Demographics	Frequency	Percentage
Gender		
**Female**	168	14.5
**Male**	994	86.5
Age		
**22–31 years**	339	29.2
**32–41 years**	282	24.3
**42–51 years**	255	21.9
**52–61**	215	18.5
**More than 61 years**	71	6.1
Marital status		
**Married**	972	83.6
**Unmarried**	173	14.9
**Separated/Widow**	17	1.5
Education		
**No education**	208	17.9
**Islamic education only**	148	12.5
**Primary**	176	15.1
**SSC**	252	21.7
**FA/FSC**	238	20.5
**BA/BSc**	75	6.5
**MA/MSc**	65	5.6
Occupation		
**Not employed**	271	23.3
**Government employee**	186	16.0
**Private job**	209	18.0
**Self-employee**	384	33.0
**House wife**	50	4.3
**Student**	62	5.3
House hold income		
**Not wanted to share**	446	38.4
**Less than 10,000**	79	6.8
**10,000–20,000**	121	10.4
**20,001–30,000**	390	33.6
**More than 30,000**	126	10.8
Locality		
**Rural**	516	44.4
**Urban**	646	55.6

### 3.2 Purchase of prescription

Table 2 shows purchase of prescription. The majority, n = 699 (60.2%), of respondents purchased medicine on prescription, and those without prescription accounted for n = 463 (39.8%), while the frequency of purchasing medicine n = 570 (49.1%) on a routine basis for refilling prescriptions.

**TABLE 2:** Purchase of prescription.

Variable	Frequency	Percentage
Purchase on prescription or without		
**No prescription**	463	39.8
**On Prescription**	699	60.2
Frequency of purchase		
**When I get disease**	570	49.1
**Routine basis refilling**	592	50.9

### 3.3 Disease prevalence for purchase of medicine

Table 3 shows disease prevalence for purchase of medicine. It was found that the majority of the diseases that accounted for more than 70% of prescription purchases were GIT problems (11.1%), pain (9.7%), hypertension (8.6%), malaria (7.4%), respiratory disease (7.2%), cold fever (7.2%), weakness (7%), UTI (5.8%), TB (4.9%), and RTA (4.5%). The rest of the diseases were categorized as other (27.3%).

**TABLE 3:** Disease prevalence for purchase of medicine.

Disease type	Frequency	Percentage
GIT problem	128	11.1
Pain	108	9.7
Hypertension	100	8.6
Malaria	86	7.4
Respiratory diseases	84	7.2
Cold fever	83	7.2
Weakness	81	7
UTI	67	5.8
T B	57	4.9
RTA	52	4.5
Other	316	27.3

### 3.4 Knowledge of pharmaceutical products and their provided information

Table 4 shows knowledge of pharmaceutical products and their provided information. Most respondents, 397 (34.2%), had never thought that all drugs dispensed/prescribed should have important information given by the manufacturer on the bottle/box or strips regarding their use. Most respondents, 612 (52.7%), always checked the information/instructions given by the manufacturer on the bottle/box/strips. The maximum number of respondents, 708 (60.9%), negated that information/instructions were easily readable. Most respondents, 811 (69.8%), never understood this information or instructions. Most respondents, 128 (11.0%), did not get information in Urdu. Most respondents, 742 (63.9%), always wanted to get information in Urdu.

**TABLE 4:** Knowledge of pharmaceutical products and their provided information.

Questions	Always	Never	Sometimes	Never think about it
All drugs which are dispensed/prescribed should have important information given by manufacturer on the bottle/box/strips with regards to its use?	291 (25.0%)	397 (34.2%)	149 (12.8%)	325 (28.0%)
Do you check the information/instructions given by manufacturer on the bottle/box/strips?	612 (52.7%)	232 (20.0%)	130 (11.2%)	188 (16.2%)
Are these information/instructions easily readable?	278 (23.9%)	708 (60.9%)	111 (9.6%)	65 (5.6%)
Do you understand these information/instructions?	67 (5.8%)	811 (69.8%)	228 (19.6%)	56 (4.8%)
Are these information/instructions given in Urdu language?	783 (67.4%)	128 (11.0%)	163 (14.0%)	88 (7.6%)
If not in Urdu language, would you want it to be given in Urdu language?	742 (63.9%)	159 (13.7%)	93 (8.0%)	168 (14.5%)

### 3.5 Knowledge of medicines for their usage and safety

Table 5 presents data on the knowledge of medicines and their usage and safety among the respondents. Most respondents knew about the usage of medicines, including what it is used for (57.1%) and how much and how often they should be taken (49.7%). However, a significant portion of respondents lacked knowledge about other aspects, such as when not to use the medicine (17.9%), potential side effects (7.1%), and the expiry date (22.2%).

**TABLE 5:** Knowledge of medicines for their usage and safety.

Questions	Yes	No	Sometimes	Don’t know
What is this medicine used for?	663 (57.1%)	235 (20.2%)	56 (4.8%)	208 (17.9%)
How much and how often should the medicine be taken, and how long the course of treatment will last	577 (49.7%)	257 (22.1%)	108 (9.3%)	220 (18.9%)
When this medicine should not be used?	208 (17.9%)	794 (68.3%)	77 (6.6%)	83 (7.1%)
What other medicines or food should be avoided while taking this medicine	633 (54.5%)	232 (20.0%)	142 (12.2%)	155 (13.3%)
How should the medicine be stored?	562 (48.4%)	272 (23.4%)	106 (9.1%)	222 (19.1%)
Any risks to the mother and the fetus or the infant from the use of the medicine during pregnancy or breast-feeding	261 (22.5%)	740 (63.7%)	67 (5.8%)	94 (8.1%)
Information on in-use shelf-life after dilution, reconstitution, or first opening, expiry	506 (43.5%)	255 (21.9%)	147 (12.7%)	254 (21.9%)
Knowledge about potential side effects of a medicine	83 (7.1%)	794 (68.3%)	77 (6.6%)	208 (17.9%)
Are you able to read the expiry date present on bottle/box/strip?	258 (22.2%)	671 (57.7%)	178 (15.3%)	55 (4.7%)

### 3.6 Knowledge regarding medication adverse drug reactions (ADRs)

Table 6 presents the study participants’ responses on their knowledge and behavior towards medication information. About 43.8% of the respondents reported always reading the patient information leaflet, while 53.3% found it difficult to understand. Only 9.6% of respondents knew that an adverse drug reaction (ADR) was an unexpected reaction after taking the normal dose, and 66.7% reported never asking or searching about their medication’s ADR. Of those who searched, 21.3% used the Internet, while 69.2% did not search at all.

**TABLE 6:** Knowledge regarding medication ADRs.

Questions	Options	F (%)
Do you read the patient information leaflet for medicines?	Always	509 (43.8%)
	Sometimes	451 (38.8%)
	Never	202 (17.4%)
Do you find the patient information leaflet difficult to understand?	Yes	619 (53.3%)
	No	377 (32.4%)
	Sometimes	166 (14.3%)
What does an adverse drug reaction (side effect/ADR) mean?	Any effect from the medication	204 (17.6%)
	Unexpected reaction after taking the normal dose	112 (9.6%)
	Expected reaction after taking the normal dose	142 (12.2%)
	I do not know	704 (60.6%)
Do you ask or search about your medication’s ADR?	Always	56 (4.8%)
	Sometimes	331 (28.5%)
	Never	775 (66.7%)
Which of the following resources do you use to search or ask about ADR?	Asking the physician	
	Asking the pharmacist	58 (5.0%)
	Internet	53 (4.6%)
	Patient Information Leaflet (PIL)	247 (21.3%)
	I do not search about it	804 (69.2%)

### 3.7 Knowledge score

Table 7 shows knowledge score. The scoring criteria have already been discussed in the methodology section. Most respondents (598 (51.5%) had good knowledge of pharmaceutical products and provided information. Most respondents, 789 (67.9%), had poor knowledge of medicines’ usage and safety. Most respondents, 711 (61.2%), knew about medication ADRs.

**TABLE 7:** Knowledge score.

Variable	Good knowledge	Poor knowledge
Knowledge of pharmaceutical products and their provided information	598 (51.5%)	564 (48.5%)
Knowledge of medicines for their usage and safety	373 (32.1%)	789 (67.9%)
Knowledge Regarding Medication ADRs	451 (38.8%)	711 (61.2%)

### 3.8 Bivariate analysis

#### 3.8.1 Bivariate analysis of knowledge of pharmaceutical products and their provided information

Table 8 shows bivariate analysis. Bivariate analysis revealed that the mean score for knowledge of pharmaceutical products and their provided information was higher in patients up to 41 years old, males, employed, educated, resided in urban populations, and purchased the medicine on prescription. Age, education, and medicine purchase were significantly associated with knowledge of pharmaceutical products and their provided information (*p* < 0.01).

**TABLE 8:** Bivariate analysis.

Variables	Knowledge of pharmaceutical products and their provided information			Knowledge of medicines for their usage and safety			Knowledge regarding medication ADRs		
	Mean (SD)	CI (95%)	*p* Values	Mean (SD)	CI (95%)	*p* Values	Mean (SD)	CI (95%)	*p* Values
**Gender**									
Female	7.345 (2.047)	(7.0244–7.6581)	0.233	6.202 (3.62)	(5.6495–6.8044)	0.233	7.577 (3.723)	(7.0176–8.1739)	0.343
Male	7.464 (2.111)			6.362 (3.77)			7.726 (3.706)		
**Age**									
Up to 41 Years	7.484 (2.074)	(7.3239–7.6487)	0.003	6.281 (3.796)	(5.9750–6.5938)	0.001	8.038 (3.757)	(7.7514–8.3407)	0.006
More than 41 Years	7.404 (2.134)			6.408 (3.691)			7.321 (3.614)		
**Education**									
No Education	7.081 (1.984)	(6.8308–7.3506)	0.005	6.009 (3.643)	(5.4976–6.5398)	0.009	7.605 (3.651)	(7.0995–8.1116)	0.006
Education	7.527 (2.119)			6.410 (3.768)			7.726 (3.721)		
**Occupation**									
Unemployed	7.436 (2.159)	(7.2948–7.5827)	0.387	6.291 (3.891)	(5.7890–6.7578)	0.02	7.652 (3.729)	(7.3925–7.9042)	0.005
Employed	7.483 (1.904)			6.535 (3.706)			7.878 (6.635)		
**Locality**									
Urban	7.519 (2.104)	(7.3340–7.7139)	0.132	6.455 (3.720)	(5.8550–6.5278)	0.454	7.945 (3.775)	(7.6297–8.2737)	0.346
Rural	7.390 (2.100)			6.193 (3.772)			7.512 (3.643)		
**Purchase on prescription**									
On Prescription	7.613 (2.061)	(7.4249–7.7973)	0.003	6.356 (3.765)	(6.0128–6.6899)	0.002	7.758 (3.690)	(7.2768–7.9703)	0.001
No Prescription	7.337 (2.123)			6.327 (3.739)			7.642 (3.735)		

**<0.01.

*<0.05.

#### 3.8.2 Bivariate analysis of knowledge of medicines for their usage and safety

Bivariate analysis revealed that the mean score for knowledge of medicines for their usage and safety was higher in patients older than 41 years, males, employed, educated, resided in urban populations, and purchased the medicine on prescription. Age, education, and purchase were significantly associated with knowledge of medicines for their usage and safety (*p* < 0.01). The occupation was significant at (*p* < 0.05).

#### 3.8.3 Bivariate analysis of knowledge regarding medication ADRs

Bivariate analysis revealed that the mean knowledge score for medication adverse drug reactions was higher in patients up to 41 years of age who were male, employed, educated, resided in urban populations, and purchased the medicine on prescription. Age, education, occupation, and purchase on prescription were significantly associated with knowledge regarding medication ADRs (*p* < 0.01).

### 3.9 Multivariate analysis

Table 9 shows multivariate analysis. Multivariate analysis revealed that purchase on prescription was a determining factor of knowledge regarding knowledge of pharmaceutical products and their provided information, medicine usage and safety, and medication ADRs. Patients who bought medicines on prescription were more likely to have better knowledge. Patients with education were more likely to have better knowledge.

**TABLE 9:** Multivariate analysis.

Variables	Model for knowledge of pharmaceutical products and their provided information			Model for knowledge of medicines for their usage and safety			Model for knowledge regarding medication ADRs		
	Coefficient **(**β) and (95% CI of β)	*p*-Value	Variance inflation factor	Coefficient **(**β) and (95% CI of β)	*p*-Value	Variance inflation factor	Coefficient **(**β) and (95% CI of β)	*p*-Value	Variance inflation factor
**Age**									
Up to 41 Years	−0.004 (−0.24, 0.16)	0.876	1.22	0.013 (−0.14, 0.26)	0.654	1.20	0.015 (0.05, 0.32)	0.746	1.32
More than 41 Years									
**Education**									
No Education	−0.014 (−0.14, 0.20)	0.832	1.28	−0.083 (−0.34, −0.08)	**0.008**	1.44	0.024 (0.15, 0.25)	**0.003**	1.58
Education									
**Occupation**									
Unemployed	----	----	----	0.009 (−0.17, 0.19)	0.565	1.35	0.012 (0.14, 0.23)	0.572	1.15
Employed									
**Purchase on prescription**									
On Prescription	0.224 (0.38, 0.63)	**<0.001**	1.43	0.223 (0.28, 0.74)	**0.003**	1.65	0.231 (0.32, 0.62)	**0.05**	1.23
No Prescription									

The bold values are Significant values (*p* < 0.05).

## 4 Discussion

In the current study, patients who visited the community pharmacy in Balochistan were asked about their knowledge of the drugs that were prescribed to them and their determinants. Our study found that only about half of the population had appropriate knowledge of pharmaceutical items and the information they offered and that knowledge of adverse drug reactions was rare. This result was in line with recent research that found that 13.2% of patients who visited an outpatient pharmacy in southwest Ethiopia were aware of the medications prescribed to them. Sri Lanka, where 17.5% of survey respondents were knowledgeable about prescription drugs (Perera T. et al., 2012; Wogayehu et al., 2020). However, comparable findings were observed in the United Kingdom, where information sources regarding medicine side effects, such as PILs, were extensive due to their accessibility. Nonetheless, many people who took traditional medications did not view them as trustworthy (O'Donovan et al., 2019).

The current study showed that consumers are purchasing medicines without a prescription. This result can be compared to another study’s conclusion that 47.2% of drugs are bought without a prescription. Prior use of the same drug in the past (Hernández-Vásquez et al., 2018). This issue is seen in Pakistan, where purchasing nearly any kind of medication without a prescription is simple. Due to self-medication and the accessibility of obtaining over-the-counter medications in large quantities, several frightening results are frequent, including resistance, addiction, withdrawal symptoms, harmful drug interactions, and hypersensitivities (Ali et al., 2020). The results corresponded with another study’s results, demonstrating a significant incidence of buying prescription drugs without a doctor’s prescription (Hernández-Vásquez et al., 2018).

The current study showed that all drugs dispensed or prescribed should have important information given by the manufacturer on the bottle, box, or strip regarding their use. This was consistent with another finding that the patient should be informed about the medication’s identity (name), dosage, administration method, frequency, length of therapy, and potential adverse effects, among other things (RHODES, 1986; Hellman-Tuitert, 1999). Patients need the right information about medicines for several reasons. This information can be given verbally or in a patient information leaflet (PIL). People cannot know which medicine is best for them until they have this knowledge, and even if they do, they cannot know how to take it properly. The name of the drug, the medicine dose, the mode of administration, the frequency of administration, the duration of therapy, the probable adverse effects, and the storage state of the medicine are all considered key parameters for safe and effective medicine usage. Adequate patient knowledge of prescribed drugs is one of the fundamental criteria for the best use of medications (Boonstra et al., 2003).

Medication guides must be provided to patients each time a prescription is filled. They are developed by the manufacturer and are FDA-approved. The current study showed that most of the time, patients check the information or instructions given by the manufacturer on the bottle, box, or strips. The safe use of all medicines depends on consumers carefully reading the label and packaging and being able to comprehend and act on the information (Shiffman et al., 2011). The current study showed that information and instructions must be easily readable. The purpose of patient information is to educate them. The written word must be read as well as understood. If patient information is to be useful, it must influence patient behavior and positively affect compliance and morbidity in whatever format it is delivered (Mayberry and Mayberry, 1996). Most patients may not be able to retain the verbal information provided by doctors for an extended period. Print materials can be useful in these cases for retaining drug usage information. Print materials can transmit basic disease or drug information (Hulka et al., 1976). Patients with chronic and complicated conditions such as diabetes, hypertension, asthma, angina, and a peptic ulcer need information about the disease and its treatments. Long-term management of these disorders requires patient self-care. Several studies have found that patients cannot control their conditions because they do not obtain the appropriate information. According to a study by Joseph et al., patients desired informational leaflets and were more concordant with their medicine when they received them from their healthcare practitioner (Nathan et al., 2007). A study showed that the package leaflet for a drug product must be readable, clear, and simple to use, and the applicant for, or holder of, a marketing authorization must provide this information (Medicines Agency HPR, 2012).

This study showed that most participants denied that information or instructions were given in Urdu. Upon subsequent concern, they wanted to get the medicine information in Urdu. The reason for this demand may be due to the literacy rate of Pakistan; it was recorded that Pakistan’s literacy rate in 2019 was 58.00%, rising 0.99% from 2018, and Pakistan continues to deal with inadequate health literacy, which frequently results in delayed disease manifestation, poor adherence to treatment, and limited awareness of health and disease prevention. In a country affected by diseases from both the developing and developed worlds, with inadequate healthcare infrastructure and low literacy levels, boosting healthcare literacy might significantly impact our people’s health and wellness (Sabzwari, 2017). This is why the Pakistani population demands that the medicine information is in their native language, Urdu. According to studies, poor health literacy leads to poor health outcomes, including misinterpreting drug instructions (Davis et al., 2006; Berkman et al., 2011).

The patient is the final link in the medication administration chain and the only person who can avoid inappropriate medication use. Educating patients about the medicines they are given is an important part of prescribing and giving out medicines. Giving patients medication information can increase adherence and patient satisfaction, reduce treatment duplication and drug interactions, and prevent potentially fatal adverse drug reactions (Davis et al., 2009). Patient medication knowledge has been characterized as knowing the drug name, purpose, administration schedule, adverse effects, or special instructions (Ascione et al., 1986).

Doctors and pharmacists in Pakistan study medicine in English, and the labeling of medications provided to patients is likewise done in English, even though the majority of patients, particularly those attending government hospitals, speak Urdu, Pashtu, or Balochi. This does not help to increase patients’ knowledge of drugs because most patients who visit hospitals, particularly those in the public sector, do not speak English well. According to research conducted in Sri Lanka, the medium of language in written material influences patients’ knowledge (Perera K. et al., 2012). Perera et al. discovered that most patients could not read and comprehend information printed in English (Perera K. et al., 2012). The importance of native language in health communication has also been demonstrated in other contexts (Wong and Wang, 2008).

One of the findings of the current study that concerned me was that knowledge about the potential side effects of medicine was not known to the majority. One of the most important predictors of successful therapy is patients’ understanding of their given medications. Poor medication knowledge can have severe ramifications, such as nonadherence and misinterpreting the significance of side effects. The findings could be comparable to how patients benefit from knowing about the potential side effects of medications so that they can notice them early and report them to their doctors. According to a Canadian study, experiencing adverse effects frequently leads to medication termination (Yee et al., 2003). A study conducted in North India found that providing relevant medicine-related information, including information about side effects, is critical to maintaining drug therapy continuation (Singh et al., 2013).

The current study showed that the majority of respondents did not know about ADR. Adverse drug reactions (ADRs) are a major source of morbidity and mortality in medication use. This was comparable to another study in which patients were also asked about adverse events that needed to be reported. The majority of respondents (67%) stated that serious adverse reactions must be recorded. Patients were correct when they stated that adverse events not described by the package leaflet (62%), as well as those covered by the pamphlet (31%), must be reported (Singh et al., 2013).

The information provided by manufacturers of pharmaceutical products is an important source of knowledge for consumers, as it can provide valuable insights into the safe and effective use of these products. However, evidence suggests that this information is often poorly understood and underutilized by consumers. This is particularly concerning given the potential health risks associated with incorrect or incomplete medication-use information.

One of the most important things for a treatment plan is for the patient to understand the drugs they are taking. Inadequate medication knowledge can have major repercussions, including nonadherence and an incorrect understanding of the importance of side effects. The study further concluded that purchase on prescription was a determining factor in knowledge regarding pharmaceutical products and their provided information, medicine usage and safety, and medication ADRs.

### 4.1 Sampling method and representativeness

Our research analyzed the general public’s awareness of medicine information, safety, and adverse drug reactions in Quetta, Pakistan. To achieve this, we carefully selected our sample based on predetermined inclusion criteria and focused on individuals who had visited community pharmacies. By targeting individuals who had sought medical advice or treatment from community pharmacies, we aimed to capture a specific subgroup of the population relevant to our research objectives. Community pharmacies play a significant role in providing accessible healthcare services to a diverse range of individuals, making them an important setting for our study. By sampling individuals who had utilized these services, we sought to gain insights into their experiences and opinions regarding healthcare provision in community pharmacies.

We recognize that achieving a representative sample can be challenging, and various factors can influence representativeness. However, our sampling approach was designed to maximize the relevance and applicability of our findings within the context of individuals seeking medical advice or treatment from community pharmacies. We strived to minimize sampling bias by employing specific inclusion criteria and targeting individuals who had visited community pharmacies, ensuring relevance and representativeness to our research objectives. While our sampling method may not capture the perspectives of the entire population, it provides valuable insights into a specific subgroup of individuals who actively seek healthcare services from community pharmacies. By focusing on this group, we can shed light on their experiences and opinions, contributing to a better understanding of healthcare provision in community pharmacy settings.

In summary, our study’s sampling approach aimed to obtain a representative sample of individuals seeking medical advice or treatment from community pharmacies. We carefully selected respondents based on predetermined inclusion criteria to capture insights from a relevant population subgroup. While acknowledging the limitations inherent in any sampling method, we believe that our approach provides valuable insights that contribute to understanding healthcare provision in community pharmacy settings.

## 5 Conclusion

Based on the study conducted to evaluate people’s awareness regarding the use of medicines, safety, and Adverse Drug Reactions (ADRs), it can be concluded that there is a need for greater awareness and education in this area. The study showed that a significant proportion of the population lacked basic knowledge about medicines, their uses, and the potential risks associated with their use. This lack of awareness can lead to the inappropriate use of medications, which can increase the risk of adverse drug reactions.

Self-medication and purchasing prescription drugs without a valid prescription are common practices in certain areas of Pakistan, particularly those with limited access to healthcare resources. These practices pose a serious risk to the health of individuals, as prescription drugs may not be suitable for their condition and may have adverse effects, leading to complications.

To address this issue, increasing awareness among the general public about the risks of self-medication and the importance of obtaining a valid prescription from a licensed healthcare provider before purchasing prescription drugs is crucial. Regulatory bodies and the government must also implement strict regulations and laws to prevent the sale of prescription drugs without a valid prescription and enforce penalties for those who engage in such practices. In conclusion, raising awareness and implementing effective regulations are necessary to ensure the safe and appropriate use of prescription drugs in Pakistan. Individuals must seek medical advice and obtain a valid prescription before purchasing prescription drugs and only purchase medications from licensed pharmacies to avoid the risk of counterfeit or adulterated drugs.

## 6 Recommendations

- Health Education Programs: Implementing health education programs in schools and communities can help increase medication knowledge.
- Public Health Campaigns: Developing public health campaigns to raise awareness about medication safety and proper use can help increase consumer knowledge.
- Healthcare Provider Training: Training healthcare providers about medication education and counseling can improve patient communication and education.
- Regulatory Oversight: Ensuring that regulatory agencies in Balochistan are adequately staffed and funded to oversee the safety and efficacy of medications can help prevent harmful outcomes. Regulatory agencies in Balochistan should be adequately staffed and funded to oversee the safety and efficacy of medications is a valid point and warrants further discussion. The role of regulatory bodies in Balochistan is critical in ensuring that medications are safe and effective for use by consumers. These regulatory bodies have the authority to demand improvements in the information provided by pharmaceutical product manufacturers to safeguard consumers’ health and wellbeing.
- Increased Access to Information: Providing access to reliable sources of information about medications can increase knowledge and improve proper use.
- Overall, improving knowledge among consumers regarding medication in Balochistan requires a multi-faceted approach that addresses cultural, social, and economic factors contributing to low knowledge levels.

## Data Availability

The original contributions presented in the study are included in the article/Supplementary Material, further inquiries can be directed to the corresponding author.
