# Supplementary material for: Public awareness regarding the manufacturer provided information about medicine usage, safety, and adverse drug reactions in Balochistan, Pakistan
Source: Front Pharmacol. 2023 Jul 20;14:1190741. doi: 10.3389/fphar.2023.1190741 (PMC10399575; doi:10.3389/fphar.2023.1190741)
Supplement: Supplementary file 1 [file Table1.DOCX]

**Supplementary file 1. Scoring of the Questionnaire**

| **Section 1:**  **Knowledge of pharmaceutical products and their provided information**  *on a Scale of 0 to 14*  *(0 = Poor Knowledge, 14 = Good Knowledge)* | **Options** | **Score** |
| --- | --- | --- |
| All drugs which are dispensed/prescribed should have important information given by manufacturer on the bottle/box/strips with regards to its use? | Yes  No  Sometimes  Don’t know | 2  0  1  0 |
| Do you check the information/instructions given by manufacturer on the bottle/box/strips? | Yes  No  Sometimes  Don’t know | 2  0  1  0 |
| Are these information/instructions easily readable? | Yes  No  Sometimes  Don’t know | 2  0  1  0 |
| Do you understand these information/ instructions? | Yes  No  Sometimes  Don’t know | 2  0  1  0 |
| Are these information/instructions given in Urdu language? | Yes  No  Sometimes  Don’t know | 2  0  1  0 |
| If not in Urdu language, would you want it to be given in Urdu language? | Yes  No  Sometimes  Don’t know | 2  0  1  0 |
| Are you able to read the expiry date present on bottle/box/strip? | Yes  No  Sometimes  Don’t know | 2  0  1  0 |
| **Section 2:**  **Knowledge of medicines for their usage and safety**  *on a Scale of 0 to 14*  *(0 = Poor Knowledge, 14 = Good Knowledge)* | **Options** | **Score** |
| What is this medicine used for? | Yes  No  Sometimes  Don’t know | 2  0  1  0 |
| How much and how often should the medicine be taken, and how long the course of treatment will last | Yes  No  Sometimes  Don’t know | 2  0  1  0 |
| When this medicine should not be used? | Yes  No  Sometimes  Don’t know | 2  0  1  0 |
| What other medicines or food should be avoided while taking this medicine | Yes  No  Sometimes  Don’t know | 2  0  1  0 |
| How should the medicine be stored? | Yes  No  Sometimes  Don’t know | 2  0  1  0 |
| Any risks to the mother and the fetus or the infant from the use of the medicine during pregnancy or breast-feeding | Yes  No  Sometimes  Don’t know | 2  0  1  0 |
| Information on in-use shelf-life after dilution, reconstitution, or first opening | Yes  No  Sometimes  Don’t know | 2  0  1  0 |
| **Section 3:**  **Knowledge Regarding Medication ADRs**  *on a Scale of 0 to 16*  *(0 = Poor Knowledge, 16 = Good Knowledge)* | **Options** | **Score** |
| Do you read the patient information leaflet for medicines? | Always  Sometimes  Never | 2  1  0 |
| Do you find the patient information leaflet difficult to understand? | Yes  No  Sometimes | 2  0  1 |
| What does an adverse drug reaction (ADR) mean? | - Any effect from the medication - Unexpected reaction after taking the normal dose - Expected reaction after taking the normal dose - I do not know | 0  2  0  0 |
| Do you ask or search about your medication’s ADR? | Always  Sometimes  Never | 2  1  0 |
| Which of the following resources do you use to search or ask about ADR? | Asking the physician  Asking the pharmacist  Internet  Patient Information Leaflet (PIL)  I do not search about it. | 2  2  2  2  0 |
